# Supplementary material for: Ruthenium(II) Complexes Coupled by Erianin via a Flexible Carbon Chain as a Potential Stabilizer of c-myc G-Quadruplex DNA
Source: Molecules. 2023 Feb 4;28(4):1529. doi: 10.3390/molecules28041529 (PMC9958891; doi:10.3390/molecules28041529)
Supplement: Supplementary file 1 [file molecules-28-01529-s001.zip › molecules-2189426-supplementary.pdf]

Supporting information for Ruthenium(II) Complexes Coupled by Erianin Via a Flexible Carbon Chain as a Potential Stabilizer of *c-myc* G-Quadruplex DNA

Zhixiang Wang [1], Wentao Liu [1], Guohu Li [1], Jiacheng Wang [1], Bin Zhao [*,2], Peishan Huang [1], and Wenjie Mei [*,1,3]

[1] School of Pharmacy, Guangdong Pharmaceutical University, Guangzhou 510006, China

[2] Department of Southern Pharmacy, Guangdong Jiangmen Chinese Medical, Jiangmen, 510047, China

[3] Guangdong Province Engineering Technology Centre for Molecular Probe and Bio-Medical Imaging, Guangzhou, 510006, China

[*] Correspondence: wenjiemei@126.com (W.M.); zhaobin3226794@163.com (B.Z.)

Table of Contents

1. Additional Table and Figures S3

2. MS and NMR Spectra (Figure S2-S13) S4-S9

2. Additional Table and Figures

Complex	λ_{ex}(nm)	λ_{em}(nm)	Stoke shift[c]	Φ_f
**1b**	466	590	124	0.2294
**2b**	466	590	124	0.2966
**1**	466	590	124	0.2866
**2**	466	590	124	0.3109

Table S1: The spectroscopic properties of the ruthenium(II) complexes.

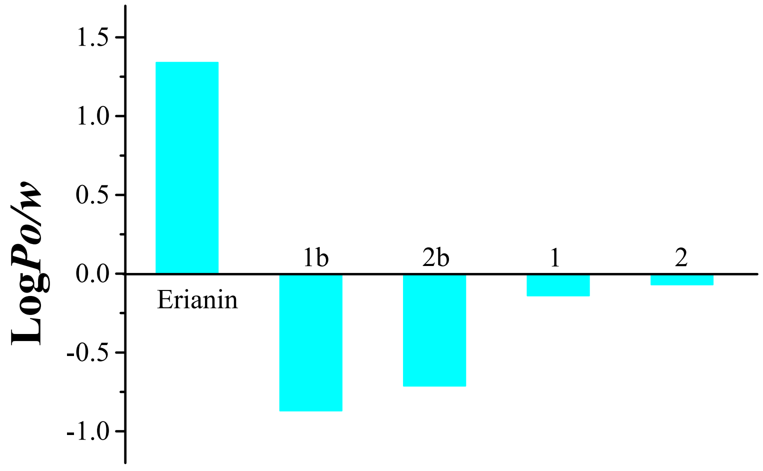

Figure S1: Octanol/water partition coefficients of ruthenium(II) complexes.

3. MS and NMR Spectra

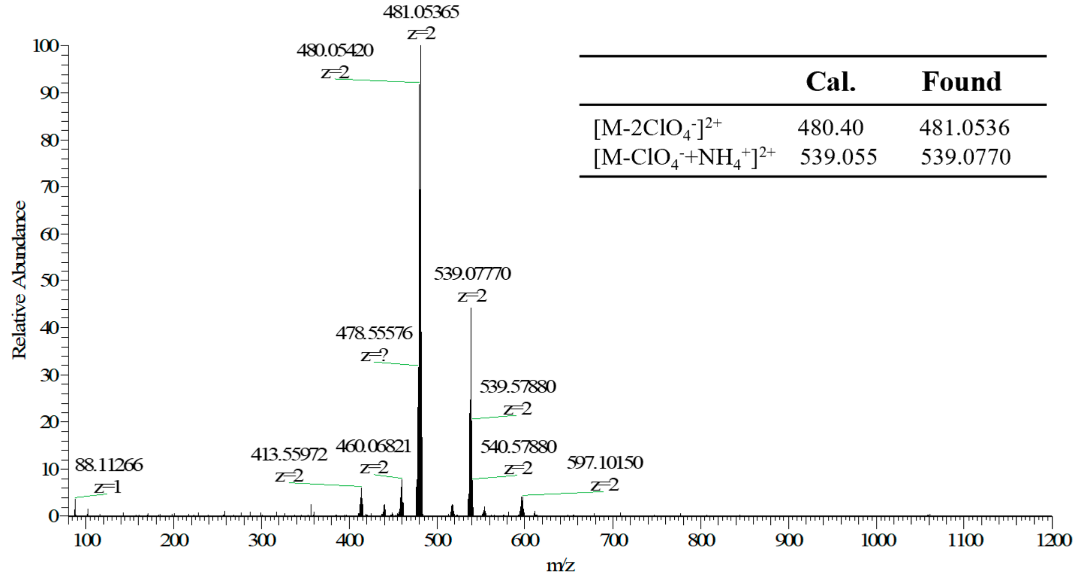

	Cal.	Found
$[M-2ClO_4^-]^{2+}$	480.40	481.0536
$[M-ClO_4^-+NH_4^+]^{2+}$	539.055	539.0770

Figure S2: ESI-MS spectrum of compound **1b**.

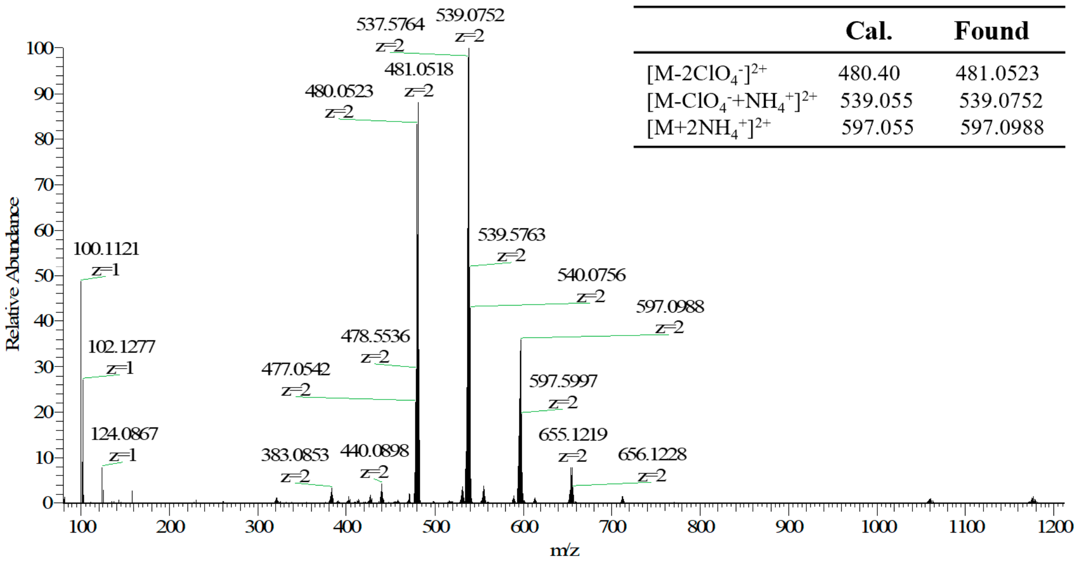

	Cal.	Found
$[M-2ClO_4^-]^{2+}$	480.40	481.0523
$[M-ClO_4^-+NH_4^+]^{2+}$	539.055	539.0752
$[M+2NH_4^+]^{2+}$	597.055	597.0988

Figure S3: ESI-MS spectrum of compound **2b.**

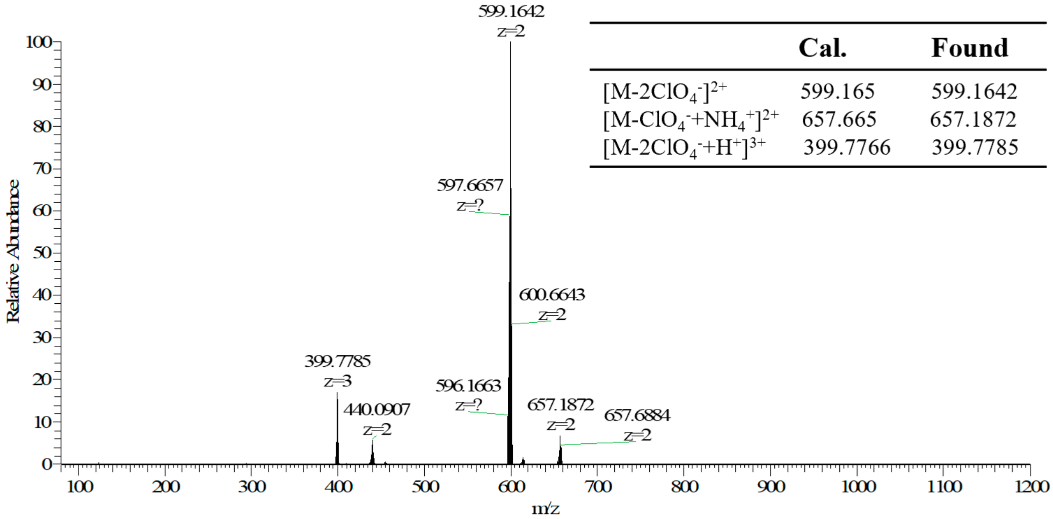

	Cal.	Found
$[M-2ClO_4^-]^{2+}$	599.165	599.1642
$[M-ClO_4^-+NH_4^+]^{2+}$	657.665	657.1872
$[M-2ClO_4^-+H^+]^{3+}$	399.7766	399.7785

Figure S4: ESI-MS spectrum of compound **1**.

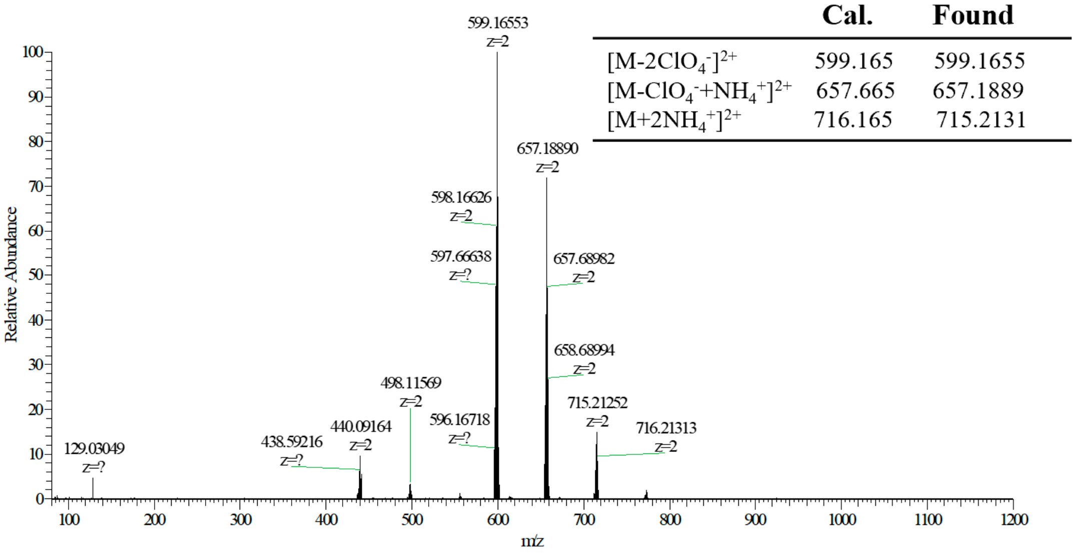

	Cal.	Found
$[M-2ClO_4^-]^{2+}$	599.165	599.1655
$[M-ClO_4^-+NH_4^+]^{2+}$	657.665	657.1889
$[M+2NH_4^+]^{2+}$	716.165	715.2131

Figure S5: ESI-MS spectrum of compound **2**.

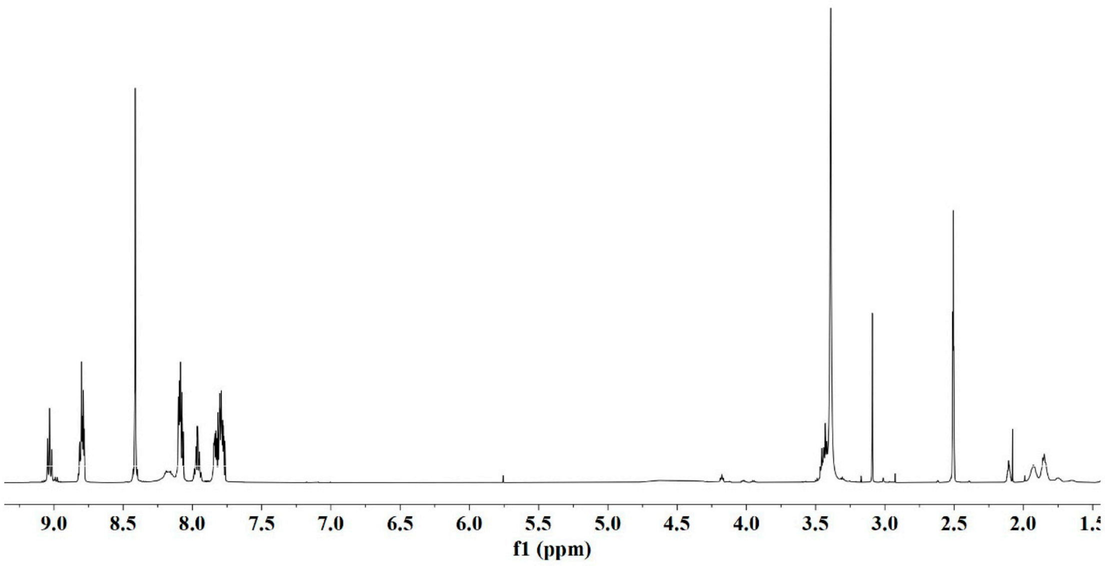

Figure S6: ^{1}H NMR spectrum of compound **1b** in DMSO-d_6.

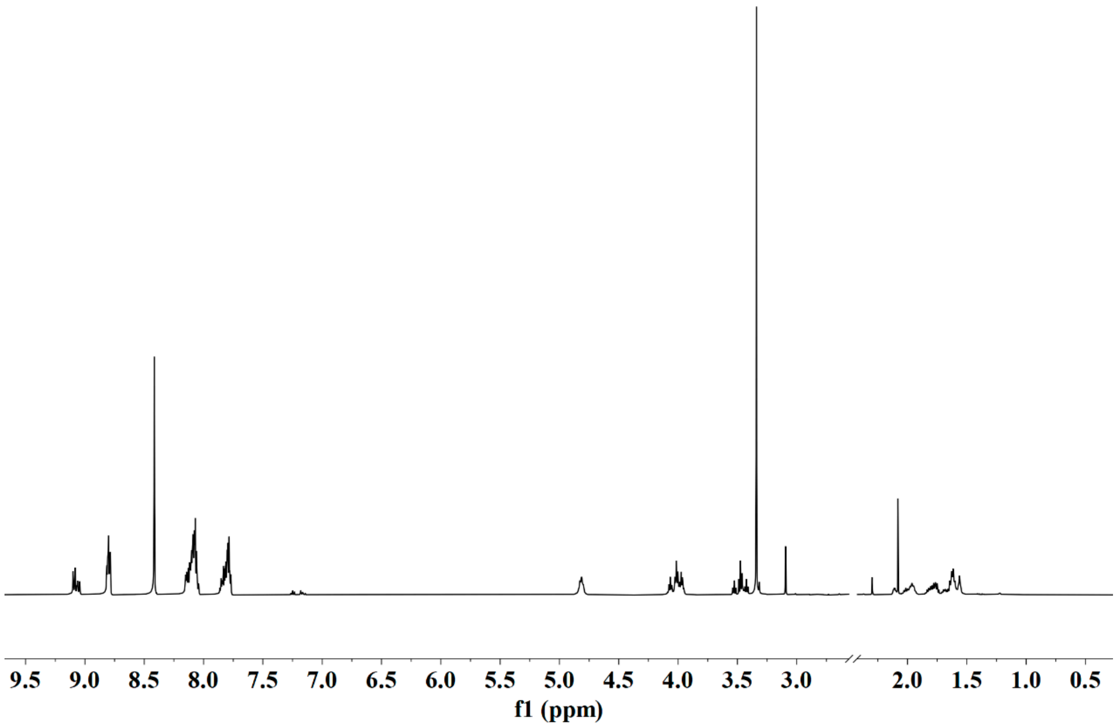

Figure S7: ^{1}H NMR spectrum of compound **2b** in DMSO-d_6.

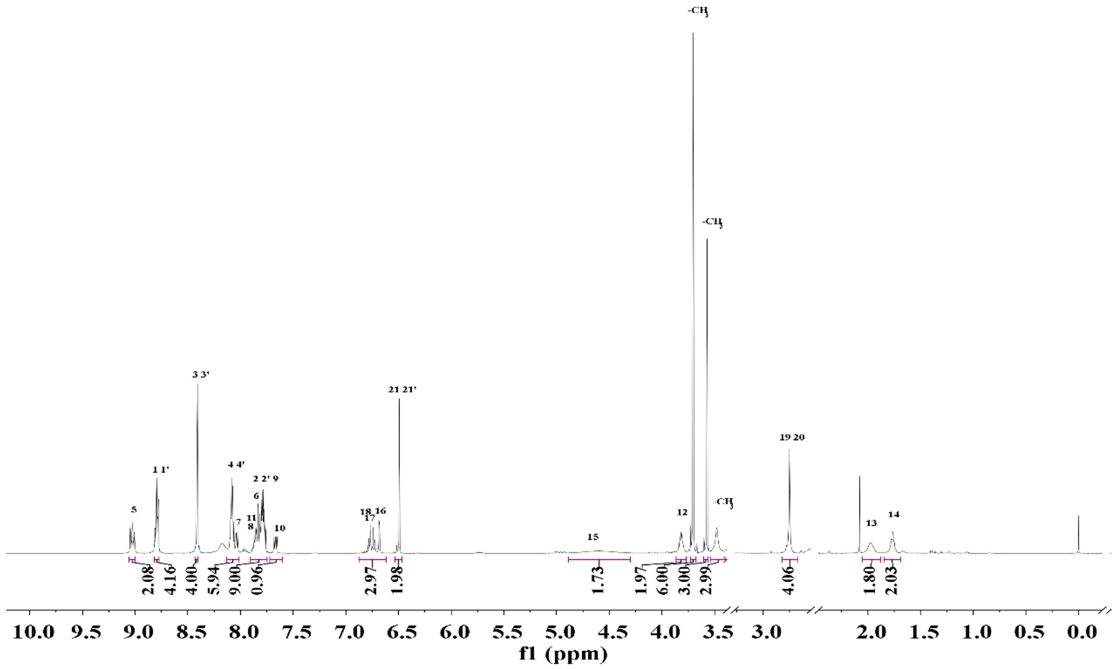

Figure S8: ^{1}H NMR spectrum of compound **1** in DMSO-*d6*.

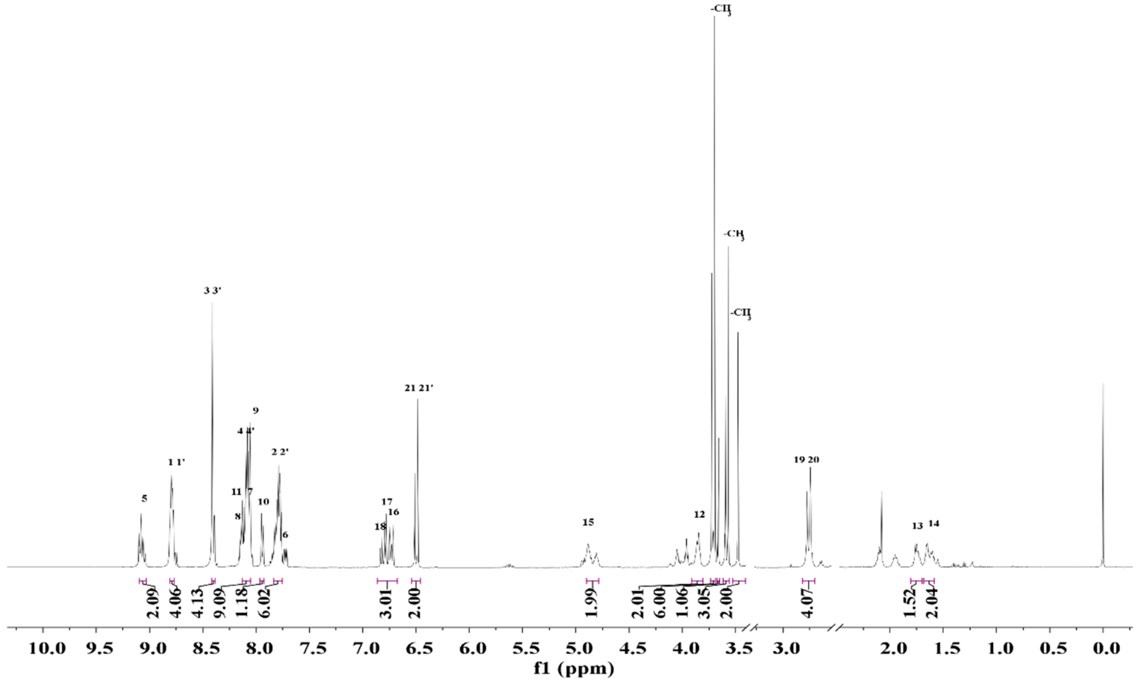

Figure S9: ^{1}H NMR spectrum of compound **2** in DMSO-*d6*.

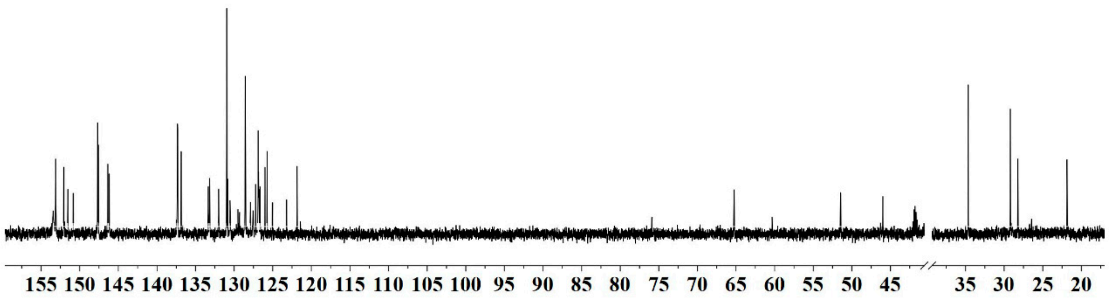

Figure S10: ^{13}C NMR spectrum of compound **1b** in DMSO-d_6.

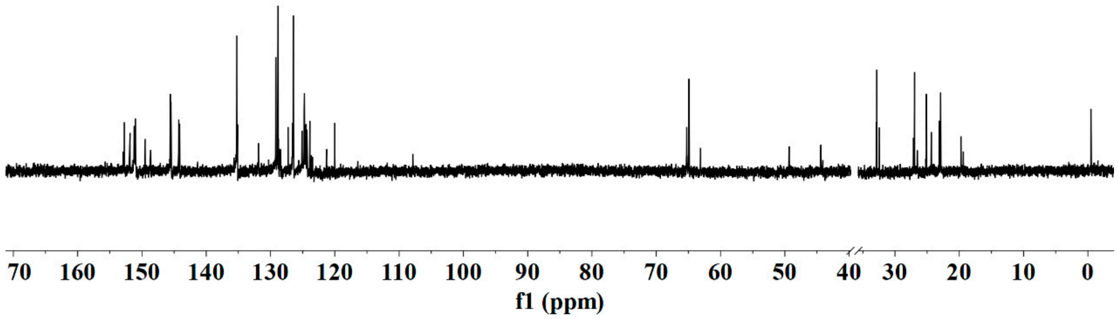

Figure S11: ^{13}C NMR spectrum of compound **2b** in DMSO-d_6.

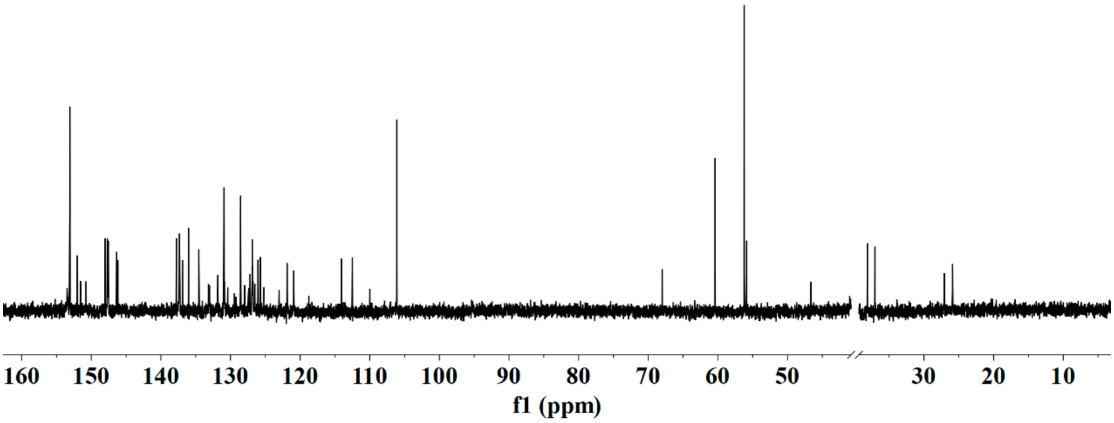

Figure S12: ^{13}C NMR spectrum of compound **1** in DMSO-d_6.

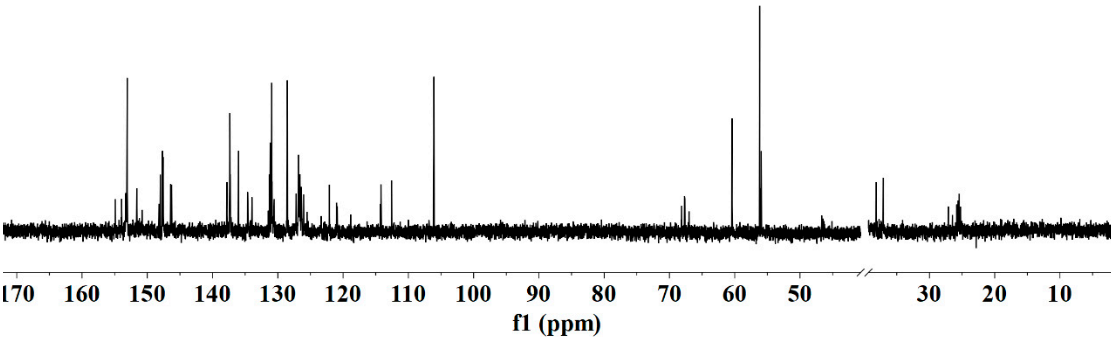

Figure S13: ^{13}C NMR spectrum of compound **2** in DMSO-d_6.
